# Supplementary material for: Structural basis for cannabinoid-induced potentiation of alpha1-glycine receptors in lipid nanodiscs
Source: Nat Commun. 2022 Aug 18;13:4862. doi: 10.1038/s41467-022-32594-5 (PMC9388682; doi:10.1038/s41467-022-32594-5)
Supplement: Supplementary file 1 — Supplementary Information [file 41467_2022_32594_MOESM1_ESM.pdf]

Supplementary Information

Structural Basis for Cannabinoid-induced Potentiation of alpha1-Glycine Receptors in Lipid Nanodiscs

Kumar et al

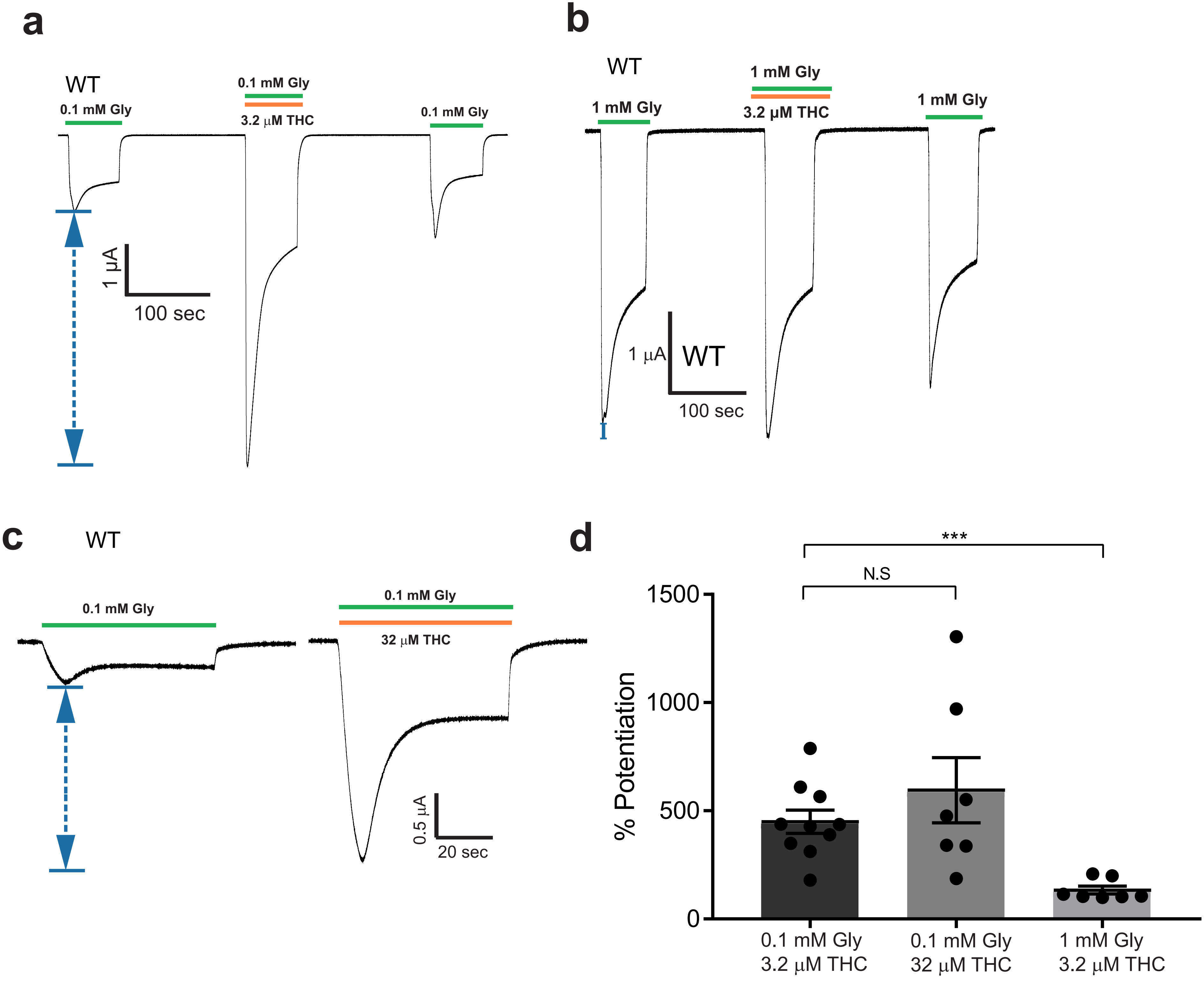
a
WT
0.1 mM Gly
0.1 mM Gly
3.2 µM THC
0.1 mM Gly
1 µA
100 sec
b
WT
1 mM Gly
1 mM Gly
3.2 µM THC
1 mM Gly
1 µA
WT
100 sec
c
WT
0.1 mM Gly
0.1 mM Gly
32 µM THC
0.5 µA
20 sec
d
***
N.S
% Potentiation
1500
1000
500
0
0.1 mM Gly
3.2 µM THC
0.1 mM Gly
32 µM THC
1 mM Gly
3.2 µM THC

Supplementary Figure 1. (a) A continuous TEVC recording of WT GlyR currents activated by 0 .1 mM glycine in the absence and presence of 3.2 μM THC. Membrane potential was held at -60 mV. (b) A representative trace from WT GlyR current recording with 1 mM glycine in the absence and presence of 3.2 μM THC. (c) A representative trace from WT GlyR current recording with 0.1 mM glycine in the absence and presence of 32 μM THC. (d) Percent potentiation is plotted as (peak of THC-glycine current / peak glycine current) x 100 for WT GlyR. Data are shown as mean ± s.e for (n) independent experiments. 0.1 mM Gly/3.2 μM THC (n = 10) 0.1 mM Gly/32 μM THC (n = 7) 1 mM Gly/3.2 μM THC (n = 7). Electrophysiology experiments were performed on independent oocytes, from multiple different surgeries. Unpaired t-test with Welch's correction *** P = 0.0024. N.S = 0.3918. Source data are available as a Source Data file.

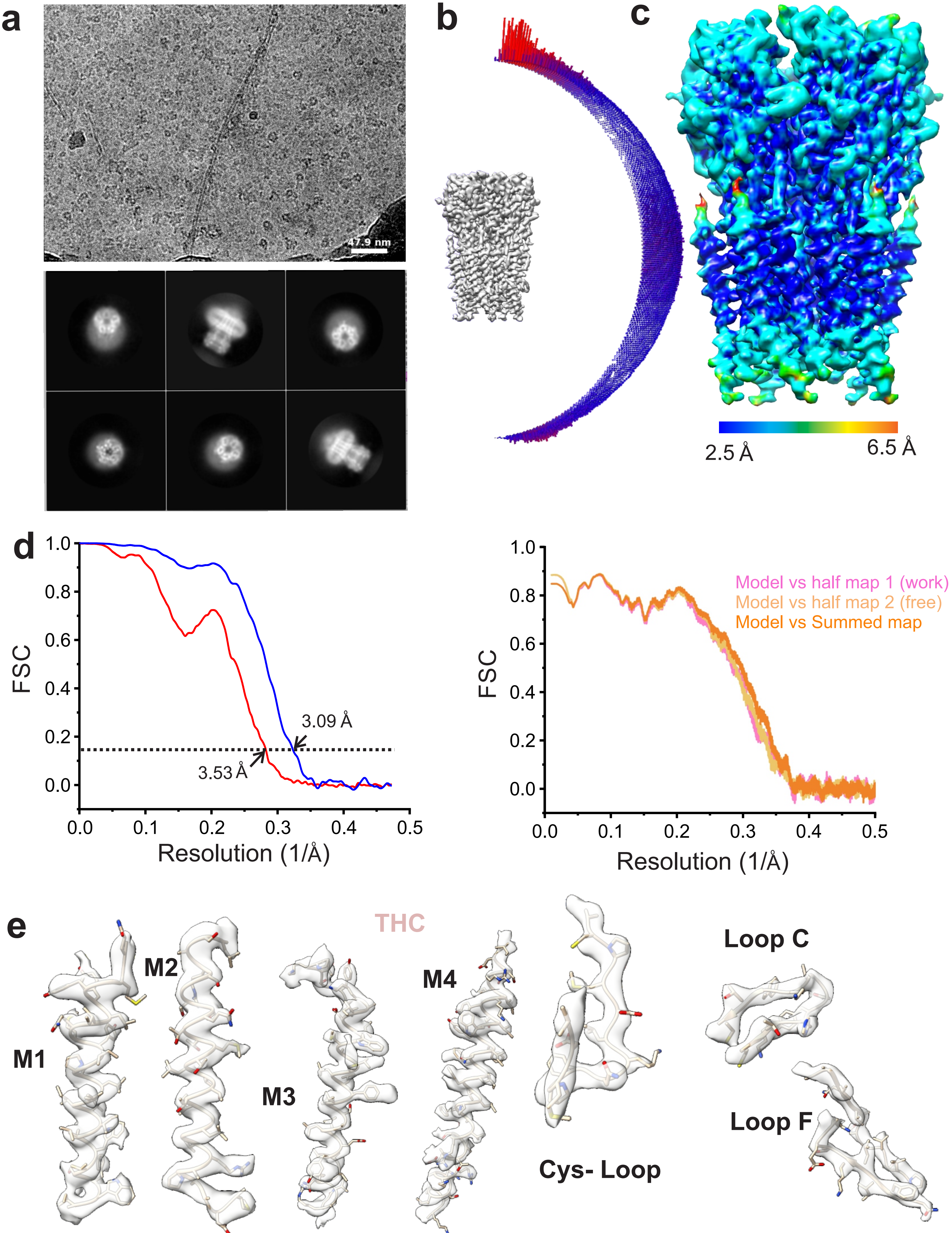

a
47.9 nm
b
c
2.5 Å
6.5 Å
d
FSC
Resolution (1/Å)
3.09 Å
3.53 Å
Model vs half map 1 (work)
Model vs half map 2 (free)
Model vs Summed map
e
THC
M1
M2
M3
M4
Cys- Loop
Loop C
Loop F

Supplementary Figure 2. Cryo-EM analysis of GlyR-THC. (a) Representative micrograph and selected 2D classes showing various particle orientations. (b) Angular distribution of particle projections for the final reconstruction used for model building. The map of the GlyR-THC complex is shown in gray. Nanodisc belts have been removed for clarity. Length of each cylinder corresponds to the number of particles at a specific Euler angle. (c) A side view of the 3D reconstruction color-coded by the local resolution determined using ResMap program algorithm[1] v1.1.5. (d) Gold standard Fourier shell correlation (FSC) curves from RELION 3.1 *(left)*. The dashed line represents an FSC of 0.143. For cross validation of model refinement, FSC curves of the refined model versus summed map (full dataset), refined model versus half map 1 (used during refinement), and refined model versus half map 2 (not used during refinement) *(right)*. (e) Map correlation of the GlyR-THC structure. Validation of various regions within each of the domains of the model (shown as cartoon with stick representation for the residues) and corresponding density map (volume) are shown here.

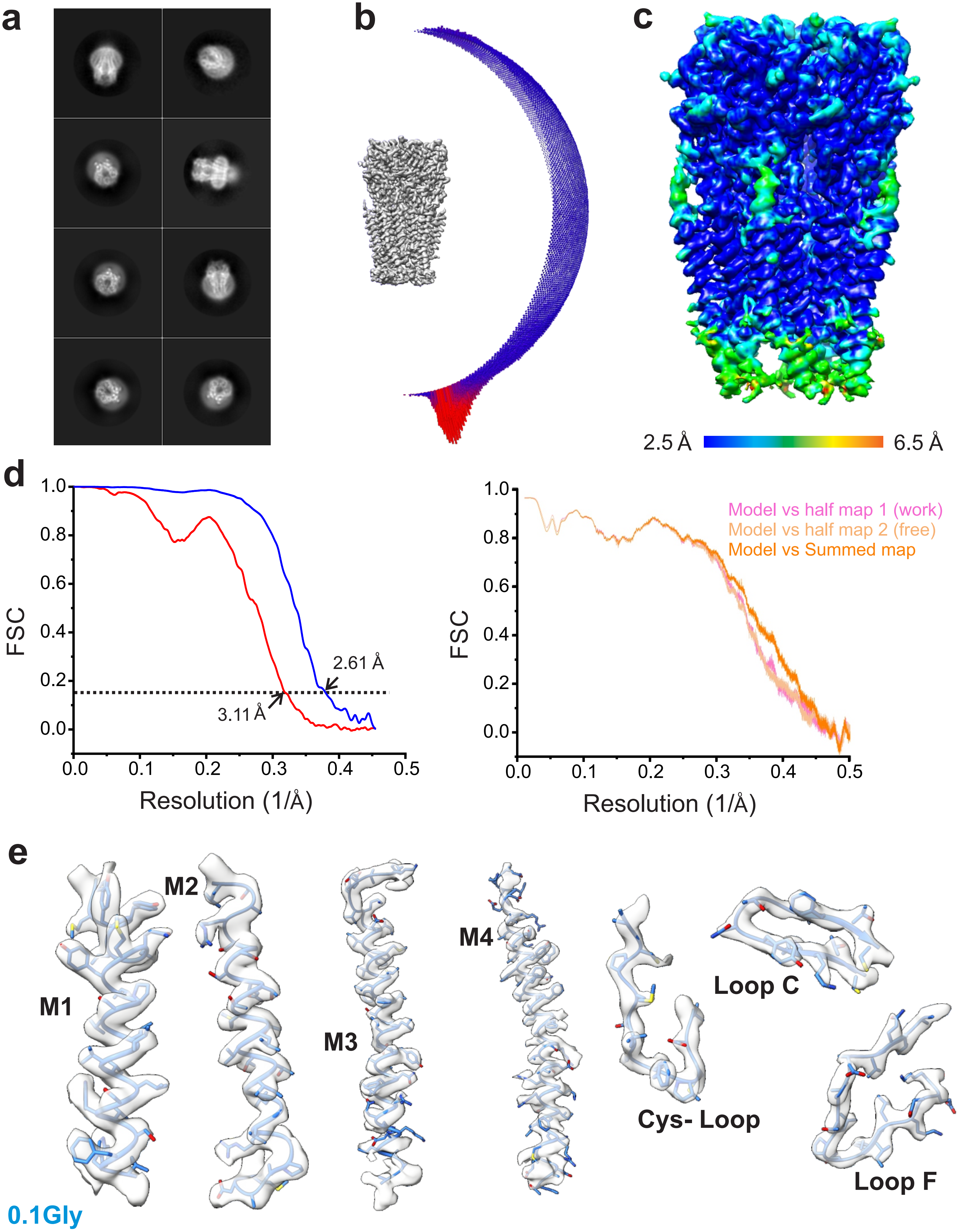

a
b
c
2.5 Å
6.5 Å
d
FSC
Resolution (1/Å)
2.61 Å
3.11 Å
Model vs half map 1 (work)
Model vs half map 2 (free)
Model vs Summed map
e
M1
M2
M3
M4
Cys- Loop
Loop C
Loop F
0.1Gly

Supplementary Figure 3. Cryo-EM analysis of GlyR-0.1gly. (a) Select 2D classes. (b) Angular distribution of particle projections for the final reconstruction used for model building. (c) 3D reconstructions color-coded by the local resolution determined using ResMap program (d) Gold standard Fourier shell correlation (FSC) curves from RELION 3.1 *(left)*. The dashed line represents an FSC of 0.143. For cross validation of model refinement, FSC curves of the refined model versus summed map (full dataset), refined model versus half map 1 *(used during refinement), and refined model versus half map 2 (not used during refinement)* (right). (e) Map correlation of GlyR-0.1gly. Validation of various regions within each of the domains of the model (shown as cartoon with stick representation for the residues) and corresponding density map (volume) are shown here.

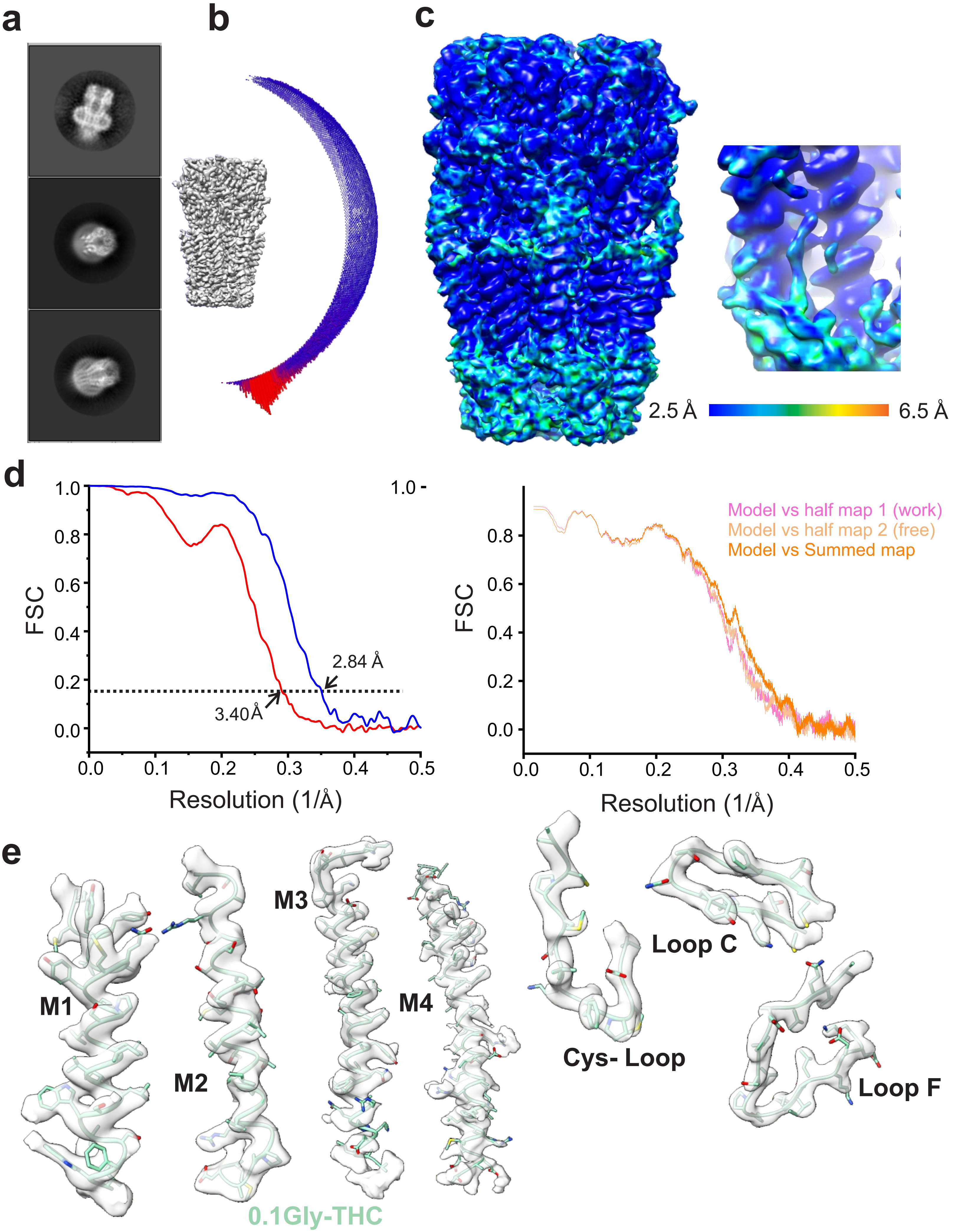

a
b
c
2.5 Å
6.5 Å
d
1.0
0.8
0.6
0.4
0.2
0.0
FSC
2.84 Å
3.40Å
0.0
0.1
0.2
0.3
0.4
0.5
Resolution (1/Å)
Model vs half map 1 (work)
Model vs half map 2 (free)
Model vs Summed map
e
M1
M2
M3
M4
Cys- Loop
Loop C
Loop F
0.1Gly-THC

Supplementary Figure 4. Cryo-EM analysis of GlyR-0.1gly-THC. (a) Select 2D classes. (b) Angular distribution of particle projections for the final reconstruction used for model building. (c) 3D reconstructions color-coded by the local resolution determined using ResMap program. *Inset* shows a zoomed in region of the THC-binding pocket (d) Gold standard Fourier shell correlation (FSC) curves from RELION 3.1 *(left)*. The dashed line represents an FSC of 0.143. For cross validation of model refinement, FSC curves of the refined model versus summed map (full dataset), refined model versus half map 1 *(used during refinement), and refined model versus half map 2 (not used during refinement)* (right). (e) Map correlation of GlyR-0.1gly-THC. Validation of various regions within each of the domains of the model (shown as cartoon with stick representation for the residues) and corresponding density map (volume) are shown here.

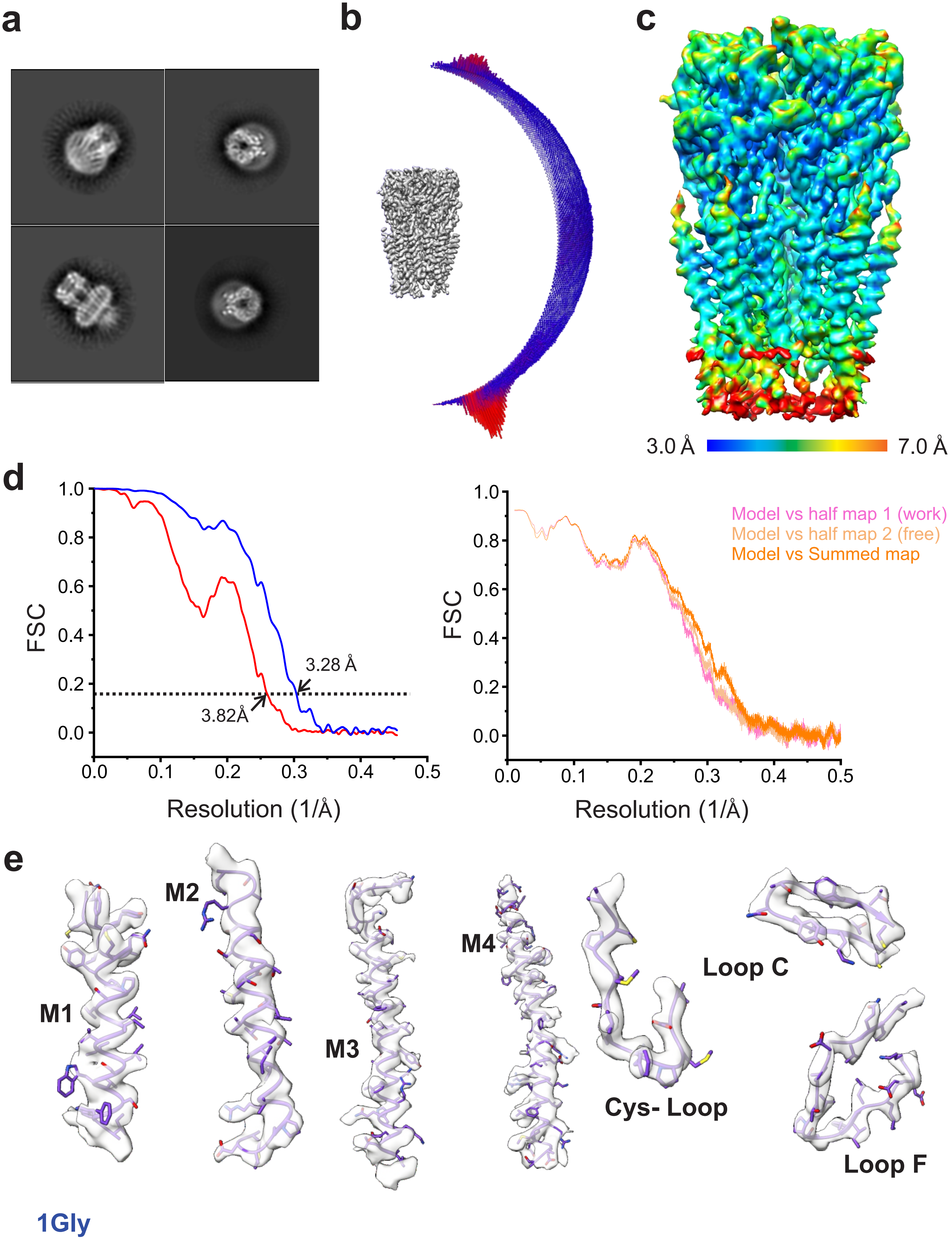
a
b
c
3.0 Å
7.0 Å
d
FSC
Resolution (1/Å)
3.28 Å
3.82Å
Model vs half map 1 (work)
Model vs half map 2 (free)
Model vs Summed map
e
M1
M2
M3
M4
Cys- Loop
Loop C
Loop F
1Gly

Supplementary Figure 5. Cryo-EM analysis of GlyR-1gly. (a) Select 2D classes. (b) Angular distribution of particle projections for the final reconstruction used for model building. (c) 3D reconstructions color-coded by the local resolution determined using ResMap program (d) Gold standard Fourier shell correlation (FSC) curves from RELION 3.1 *(left)*. The dashed line represents a n FSC of 0.143. For cross validation of model refinement, FSC curves of the refined model versus summed map (full dataset), refined model versus half map 1 *(used during refinement), and refined model versus half map 2 (not used during refinement)* (right). (e) Map correlation of GlyR-1gly. Validation of various regions within each of the domains of the model (shown as cartoon with stick representation for the residues) and corresponding density map (volume) are shown here.

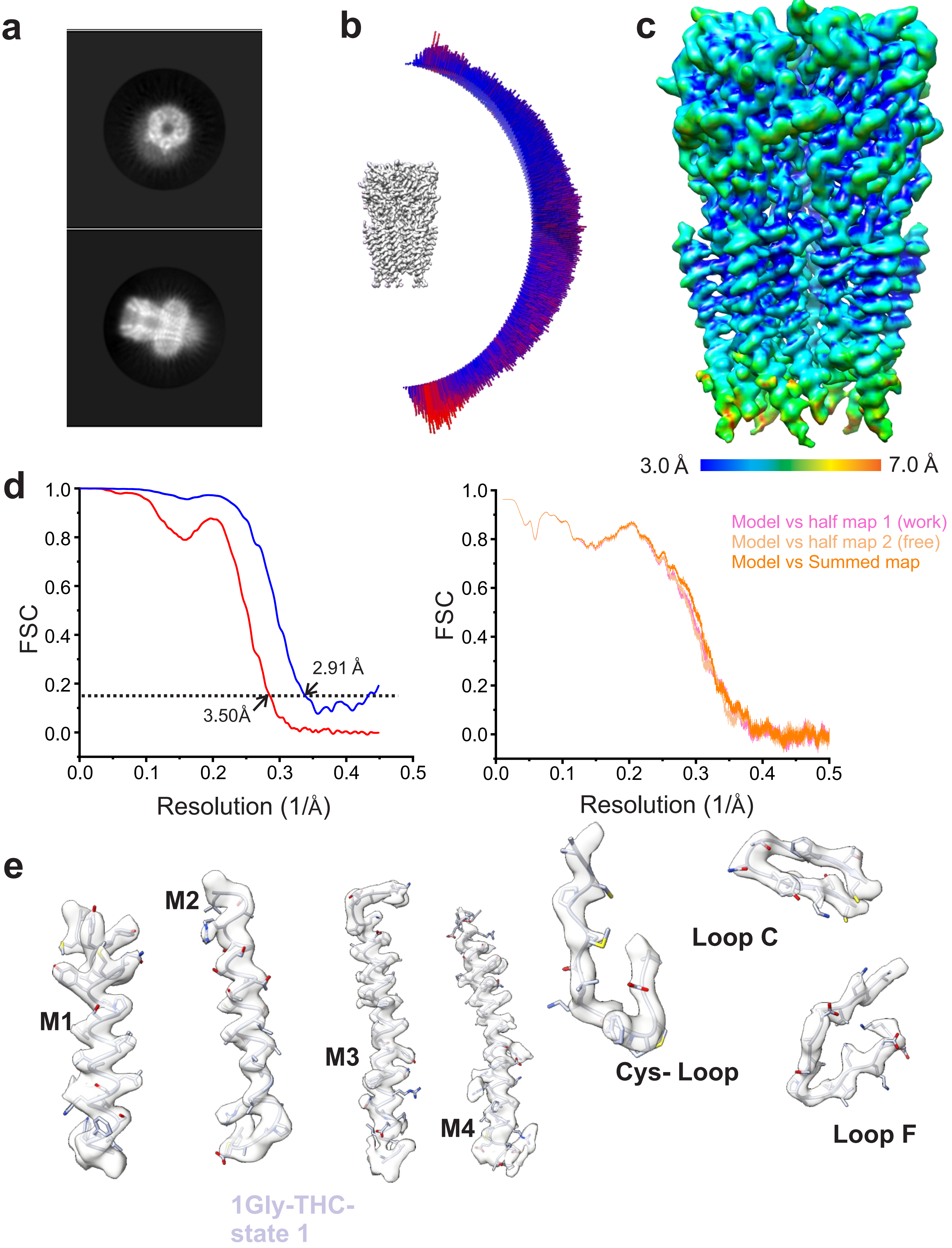
a
b
c
3.0 Å
7.0 Å
d
1.0
0.8
0.6
0.4
0.2
0.0
FSC
2.91 Å
3.50Å
0.0
0.1
0.2
0.3
0.4
0.5
Resolution (1/Å)
Model vs half map 1 (work)
Model vs half map 2 (free)
Model vs Summed map
e
M1
M2
M3
M4
Cys- Loop
Loop C
Loop F
1Gly-THC-
state 1

Supplementary Figure 6. Cryo-EM analysis of GlyR-1gly-THC-State1. (a) Select 2D classes. (b) Angular distribution of particle projections for the final reconstruction used for model building. (c) 3D reconstructions color-coded by the local resolution determined using ResMap program (d) Gold standard Fourier shell correlation (FSC) curves from RELION 3.1 *(left)*. The dashed line represents an FSC of 0.143. For cross validation of model refinement, FSC curves of the refined model versus summed map (full dataset), refined model versus half map 1 *(used during refinement), and refined model versus half map 2 (not used during refinement)* (right). (e) Map correlation of GlyR-1gly-state1. Validation of various regions within each of the domains of the model (shown as cartoon with stick representation for the residues) and corresponding density map (volume) are shown here.

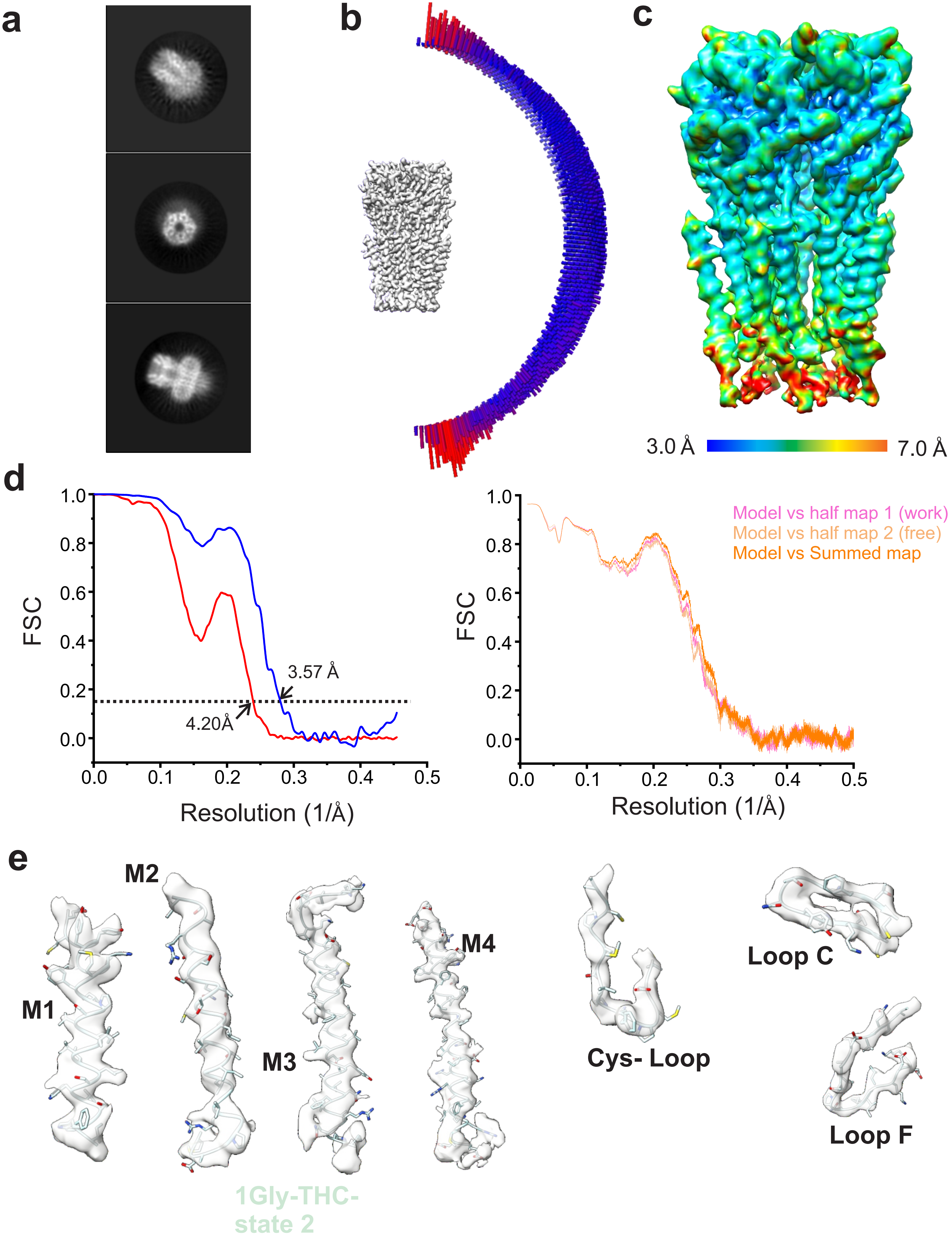
a
b
c
3.0 Å
7.0 Å
d
1.0
0.8
0.6
0.4
0.2
0.0
FSC
3.57 Å
4.20Å
0.0
0.1
0.2
0.3
0.4
0.5
Resolution (1/Å)
Model vs half map 1 (work)
Model vs half map 2 (free)
Model vs Summed map
e
M1
M2
M3
M4
Cys- Loop
Loop C
Loop F
1Gly-THC-
state 2

Supplementary Figure 7. Cryo-EM analysis of GlyR-1gly-THC-State 2. (a) Select 2D classes. (b) Angular distribution of particle projections for the final reconstruction used for model building. (c) 3D reconstructions color-coded by the local resolution determined using ResMap program (d) Gold standard Fourier shell correlation (FSC) curves from RELION 3.1 *(left)*. The dashed line represents an FSC of 0.143. For cross validation of model refinement, FSC curves of the refined model versus summed map (full dataset), refined model versus half map 1 *(used during refinement), and refined model versus half map 2 (not used during refinement)* (right). (e) Map correlation of GlyR-1gly-state2. Validation of various regions within each of the domains of the model (shown as cartoon with stick representation for the residues) and corresponding density map (volume) are shown here.

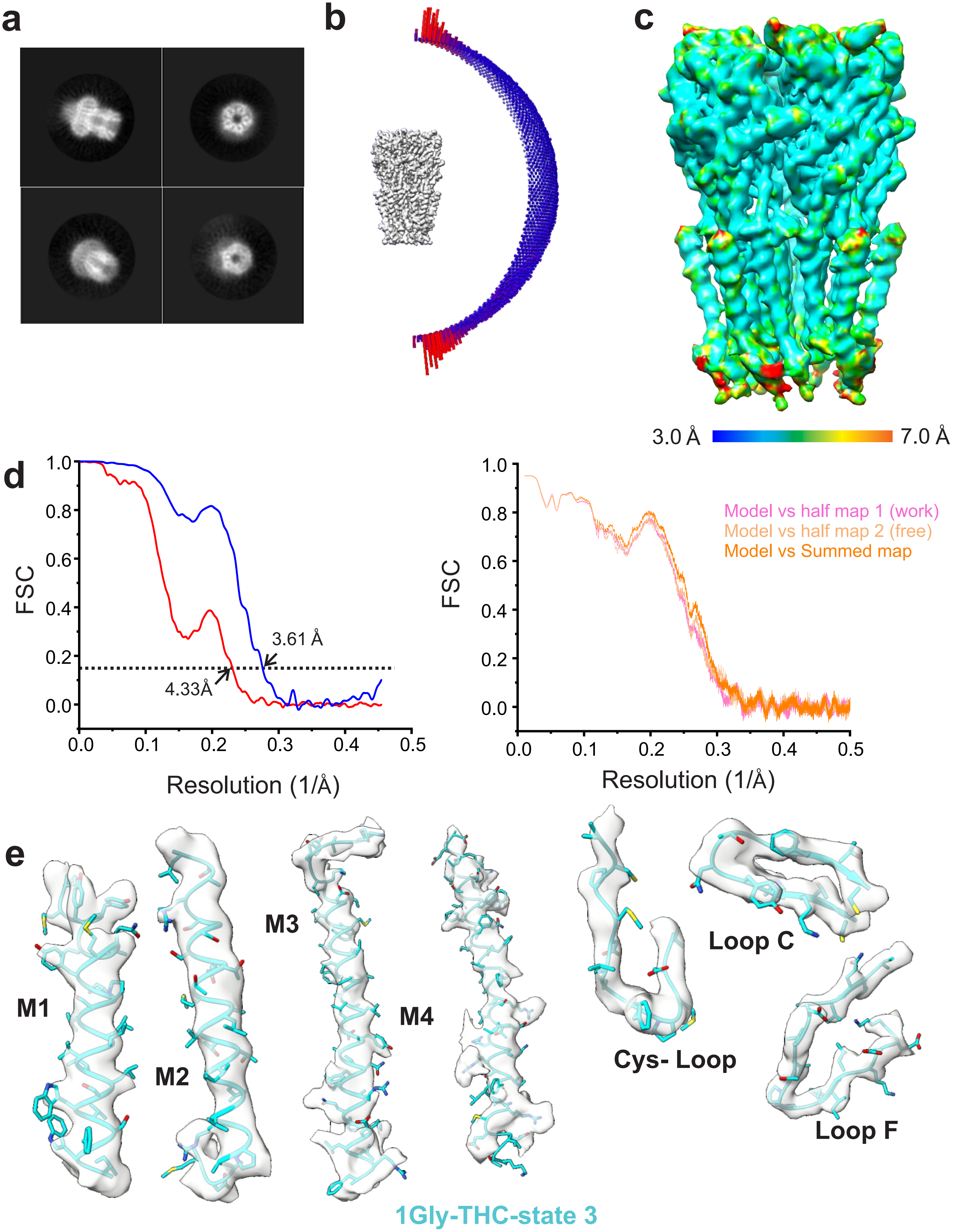

a
b
c
3.0 Å
7.0 Å
d
1.0
0.8
0.6
0.4
0.2
0.0
FSC
3.61 Å
4.33Å
0.0
0.1
0.2
0.3
0.4
0.5
Resolution (1/Å)
Model vs half map 1 (work)
Model vs half map 2 (free)
Model vs Summed map
e
M1
M2
M3
M4
Cys- Loop
Loop C
Loop F
1Gly-THC-state 3

Supplementary Figure 8. Cryo-EM analysis of GlyR-1gly-THC-State 3. (a) Select 2D classes. (b) Angular distribution of particle projections for the final reconstruction used for model building. (c) 3D reconstructions color-coded by the local resolution determined using ResMap program (d) Gold standard Fourier shell correlation (FSC) curves from RELION 3.1 *(left)*. The dashed line represents an FSC of 0.143. For cross validation of model refinement, FSC curves of the refined model versus summed map (full dataset), refined model versus half map 1 *(used during refinement), and refined model versus half map 2 (not used during refinement)* (right). (e) Map correlation of GlyR-1gly-state1. Validation of various regions within each of the domains of the model (shown as cartoon with stick representation for the residues) and corresponding density map (volume) are shown here.

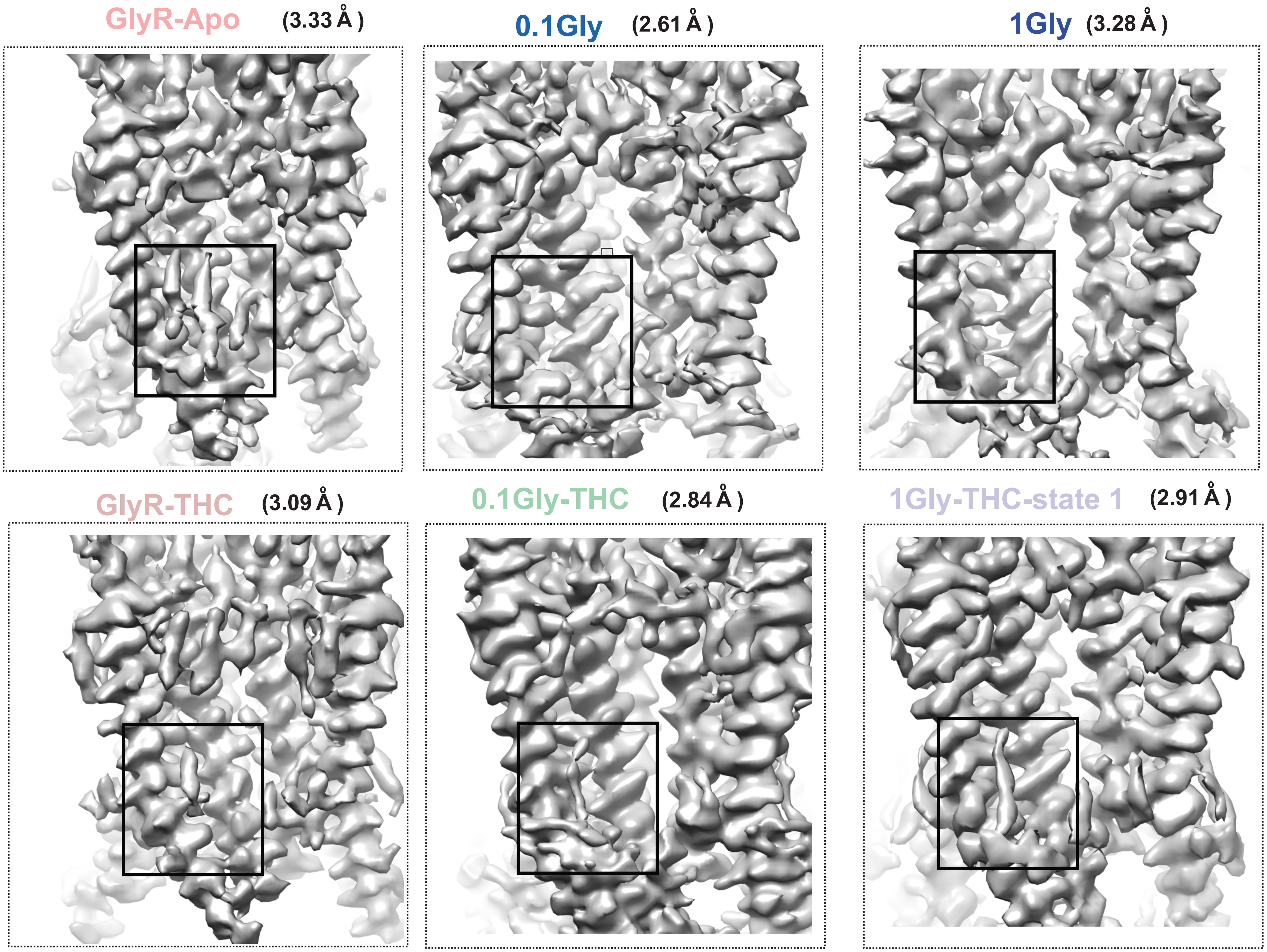
GlyR-Apo (3.33 Å)
0.1Gly (2.61 Å)
1Gly (3.28 Å)
GlyR-THC (3.09 Å)
0.1Gly-THC (2.84 Å)
1Gly-THC-state 1 (2.91 Å)

Supplementary Figure 9. Density at the THC binding pocket in the Cryo-EM 3D reconstructions in various states. THC and phospholipid density observed in various GlyR reconstructions. Shown here are RELION 3.1 postprocess maps The maps are displayed at following σ levels: GlyR-Apo (0.010), 0.1Gly (0.016), 1Gly (0.008) GlyR-THC (0.016), 0.1Gly-THC (0.004), and 1Gly-THC-state1 (0.008). Two adjacent units are highlighted for clarity. The region around THC binding pocket is indicated by a box. The nominal resolutions for each map is shown in parenthesis.

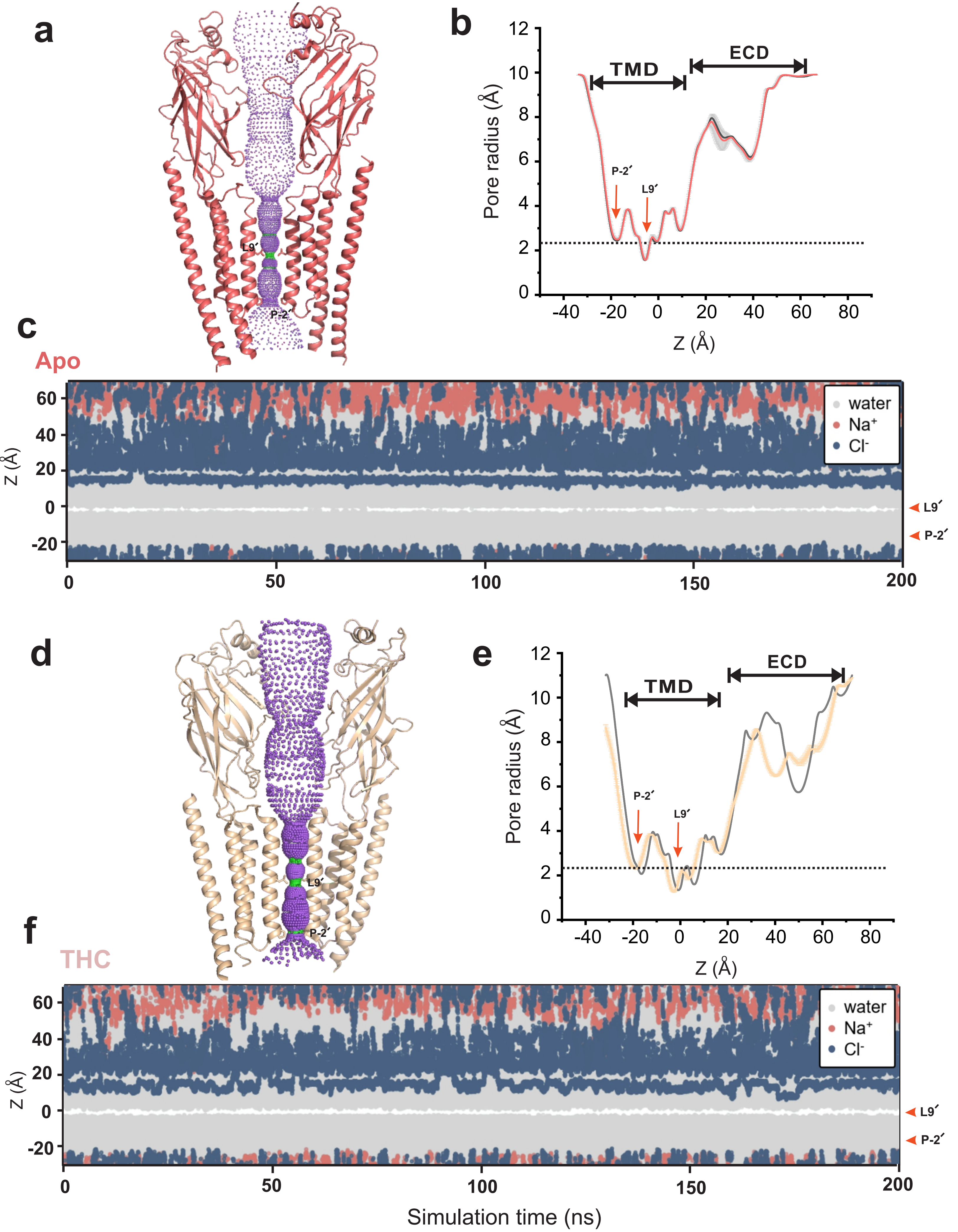

a
L9´
P-2´
b
TMD
ECD
P-2´
L9´
Pore radius (Å)
Z (Å)
c
Apo
water
Na+
Cl-
Z (Å)
L9´
P-2´
d
L9´
P-2´
e
TMD
ECD
P-2´
L9´
Pore radius (Å)
Z (Å)
f
THC
water
Na+
Cl-
Z (Å)
L9´
P-2´
Simulation time (ns)

Supplementary Figure 10. Assessment of conductance state of GlyR-Apo and GlyR-THC (a) Ion permeation pathway generated with HOLE for GlyR-Apo. For clarity, only two non-adjacent subunits are shown. Colors of the spheres represent the following pore radii: red <1.15 Å, green 1.8–2.3 Å and purple >2.3 Å (b) Mean pore radius and one-standard deviations from three independent 30 ns equilibrium simulations for GlyR-Apo structure along the central pore axis. Major constriction sites are indicated and the dotted line denotes the radius of hydrated chloride ions. The gray trace is the pore radius profile calculated from the cryo-EM structures. (c) Simulation trajectories along the pore (z)-axis of water molecules and chloride ion coordinates within 5 Å of the channel axis inside the pore of GlyR-Apo structure, in the presence of a +500 mV transmembrane potential difference (i.e., with the cytoplasmic side having a positive potential). One of five independent 200 ns replicates is shown for each structure. The energetic barriers due to the ring of Leu9′ and Pro-2′ are at z ~0 and −20 Å, respectively. (d) Ion permeation pathway generated for GlyR-THC structure. (e) Mean pore radius profiles and standard deviations averaged across three independent 30 ns equilibrium simulations for GlyR-THC. (f) Simulation trajectories along the pore (z)-axis of water molecules and chloride ion coordinates for GlyR-THC.

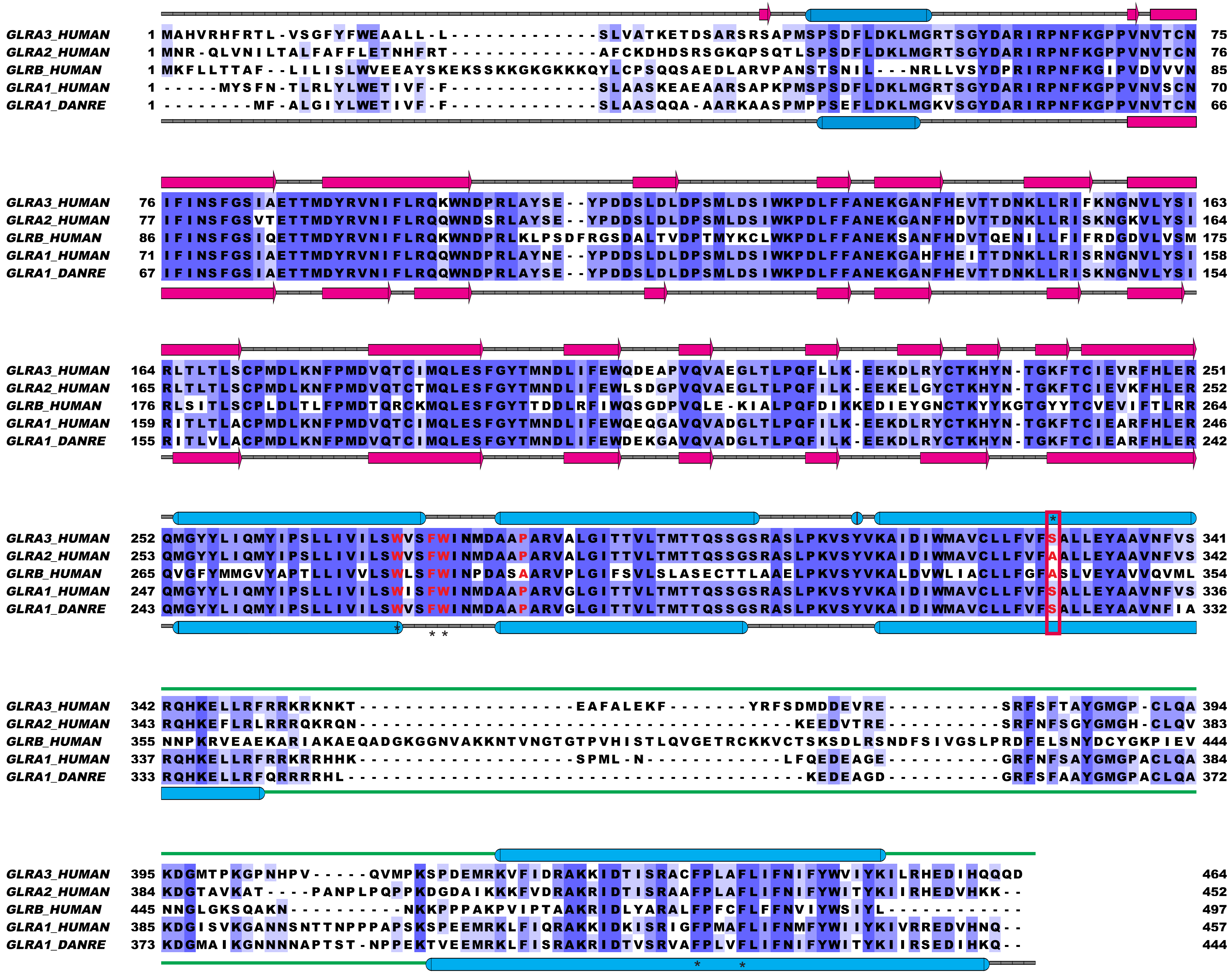

GLRA3_HUMAN 1 MAHVRHFRTL-VSGFYFWEAALL-L-------------SLVATKETDSARSRSAPMSPSDFLDKLMGRTSGYDARIRPNFKGPPVNVTCN 75
GLRA2_HUMAN 1 MNR-QLVNILTALFAFFLETNHFRT-------------AFCKDHDSRSGKQPSQTLSPSDFLDKLMGRTSGYDARIRPNFKGPPVNVTCN 76
GLRB_HUMAN 1 MKFLLTTAF--LILISLWVEEAYSKEKSSKKGKGKKKQYLCPSQQSAEDLARVPANSTSNIL---NRLLVSYDPRIRPNFKGIPVDVVVN 85
GLRA1_HUMAN 1 -----MYSFN-TLRLYLWETIVF-F-------------SLAASKEAEAARSAPKPMSPSDFLDKLMGRTSGYDARIRPNFKGPPVNVSCN 70
GLRA1_DANRE 1 --------MF-ALGIYLWETIVF-F-------------SLAASQQA-AARKAASPMPPSEFLDKLMGKVSGYDARIRPNFKGPPVNVTCN 66
GLRA3_HUMAN 76 IFINSFGSIAETTMDYRVNIFLRQKWNDPRLAYSE--YPDDSLDLDPSMLDSIWKPDLFFANEKGANFHEVTTDNKLLRIFKNGNVLYSI 163
GLRA2_HUMAN 77 IFINSFGSVTETTMDYRVNIFLRQQWNDSRLAYSE--YPDDSLDLDPSMLDSIWKPDLFFANEKGANFHDVTTDNKLLRISKNGKVLYSI 164
GLRB_HUMAN 86 IFINSFGSIQETTMDYRVNIFLRQKWNDPRLKLPSDFRGSDALTVDPTMYKCLWKPDLFFANEKSANFHDVTQENILLFIFRDGDVLVSM 175
GLRA1_HUMAN 71 IFINSFGSIAETTMDYRVNIFLRQQWNDPRLAYNE--YPDDSLDLDPSMLDSIWKPDLFFANEKGAHFHEITTDNKLLRISRNGNVLYSI 158
GLRA1_DANRE 67 IFINSFGSIAETTMDYRVNIFLRQQWNDPRLAYSE--YPDDSLDLDPSMLDSIWKPDLFFANEKGANFHEVTTDNKLLRISKNGNVLYSI 154
GLRA3_HUMAN 164 RLTLTLSCPMDLKNFPMDVQTCIMQLESFGYTMNDLIFEWQDEAPVQVAEGLTLPQFLLK-EEKDLRYCTKHYN-TGKFTCIEVRFHLER 251
GLRA2_HUMAN 165 RLTLTLSCPMDLKNFPMDVQTCTMQLESFGYTMNDLIFEWLSDGPVQVAEGLTLPQFILK-EEKELGYCTKHYN-TGKFTCIEVKFHLER 252
GLRB_HUMAN 176 RLSITLSCPLDLTLFPMDTQRCKMQLESFGYTTDDLRFIWQSGDPVQLE-KIALPQFDIKKEDIEYGNCTKYYKGTGYYTCVEVIFTLRR 264
GLRA1_HUMAN 159 RITLTLACPMDLKNFPMDVQTCIMQLESFGYTMNDLIFEWQEQGAVQVADGLTLPQFILK-EEKDLRYCTKHYN-TGKFTCIEARFHLER 246
GLRA1_DANRE 155 RITLVLACPMDLKNFPMDVQTCIMQLESFGYTMNDLIFEWDEKGAVQVADGLTLPQFILK-EEKDLRYCTKHYN-TGKFTCIEARFHLER 242
*
GLRA3_HUMAN 252 QMGYYLIQMYIPSLLIVILSWVSFWINMDAAPARVALGITTVLTMTTQSSGSRASLPKVSYVKAIDIWMAVCLLFVFSALLEYAAVNFVS 341
GLRA2_HUMAN 253 QMGYYLIQMYIPSLLIVILSWVSFWINMDAAPARVALGITTVLTMTTQSSGSRASLPKVSYVKAIDIWMAVCLLFVFAALLEYAAVNFVS 342
GLRB_HUMAN 265 QVGFYMMGVYAPTLLIVVLSWLSFWINPDASAARVPLGIFSVLSLASECTTLAAELPKVSYVKALDVWLIACLLFGFASLVEYAVVQVML 354
GLRA1_HUMAN 247 QMGYYLIQMYIPSLLIVILSWISFWINMDAAPARVGLGITTVLTMTTQSSGSRASLPKVSYVKAIDIWMAVCLLFVFSALLEYAAVNFVS 336
GLRA1_DANRE 243 QMGYYLIQMYIPSLLIVILSWVSFWINMDAAPARVGLGITTVLTMTTQSSGSRASLPKVSYVKAIDIWMAVCLLFVFSALLEYAAVNFIA 332
* * *
GLRA3_HUMAN 342 RQHKELLRFRRKRKNKT-------------------EAFALEKF-------YRFSDMDDEVRE----------SRFSFTAYGMGP-CLQA 394
GLRA2_HUMAN 343 RQHKEFLRLRRRQKRQN--------------------------------------KEEDVTRE----------SRFNFSGYGMGH-CLQV 383
GLRB_HUMAN 355 NNPKRVEAEKARIAKAEQADGKGGNVAKKNTVNGTGTPVHISTLQVGETRCKKVCTSKSDLRSNDFSIVGSLPRDFELSNYDCYGKPIEV 444
GLRA1_HUMAN 337 RQHKELLRFRRKRRHHK-------------------SPML-N-------------LFQEDEAGE----------GRFNFSAYGMGPACLQA 384
GLRA1_DANRE 333 RQHKELLRFQRRRRHL--KEDEAGD----------GRFSFAAYGMGPACLQA 372
GLRA3_HUMAN 395 KDGMTPKGPNHPV-----QVMPKSPDEMRKVFIDRAKKIDTISRACFPLAFLIFNIFYWVIYKILRHEDIHQQQD 464
GLRA2_HUMAN 384 KDGTAVKAT----PANPLPQPPKDGDAIKKKFVDRAKRIDTISRAAFPLAFLIFNIFYWITYKIIRHEDVHKK-- 452
GLRB_HUMAN 445 NNGLGKSQAKN----------NKKPPPAKPVIPTAAKRIDLYARALFPFCFLFFNVIYWSIYL------------ 497
GLRA1_HUMAN 385 KDGISVKGANNSNTTNPPPAPSKSPEEMRKLFIQRAKKIDKISRIGFPMAFLIFNMFYWIIYKIVRREDVHNQ-- 457
GLRA1_DANRE 373 KDGMAIKGNNNNAPTST-NPPEKTVEEMRKLFISRAKRIDTVSRVAFPLVFLIFNIFYWITYKIIRSEDIHKQ-- 444
* *

Supplementary Figure 11 Multiple sequence alignment of GlyR Sequence of *Danio rerio* GlyRα1 used in the cryo-EM study and electrophysiological analysis aligned to *Homo sapiens* GlyRα1, *Homo sapiens* GlyRα2, *Homo sapiens* GlyRα3 and *Homo sapiens* GlyRβ. Secondary structural elements are indicated for *Homo sapiens* GlyRα3 (above) and *Danio rerio* GlyRα1 (below) the sequence. Green line denotes the residues not included in the structural models.

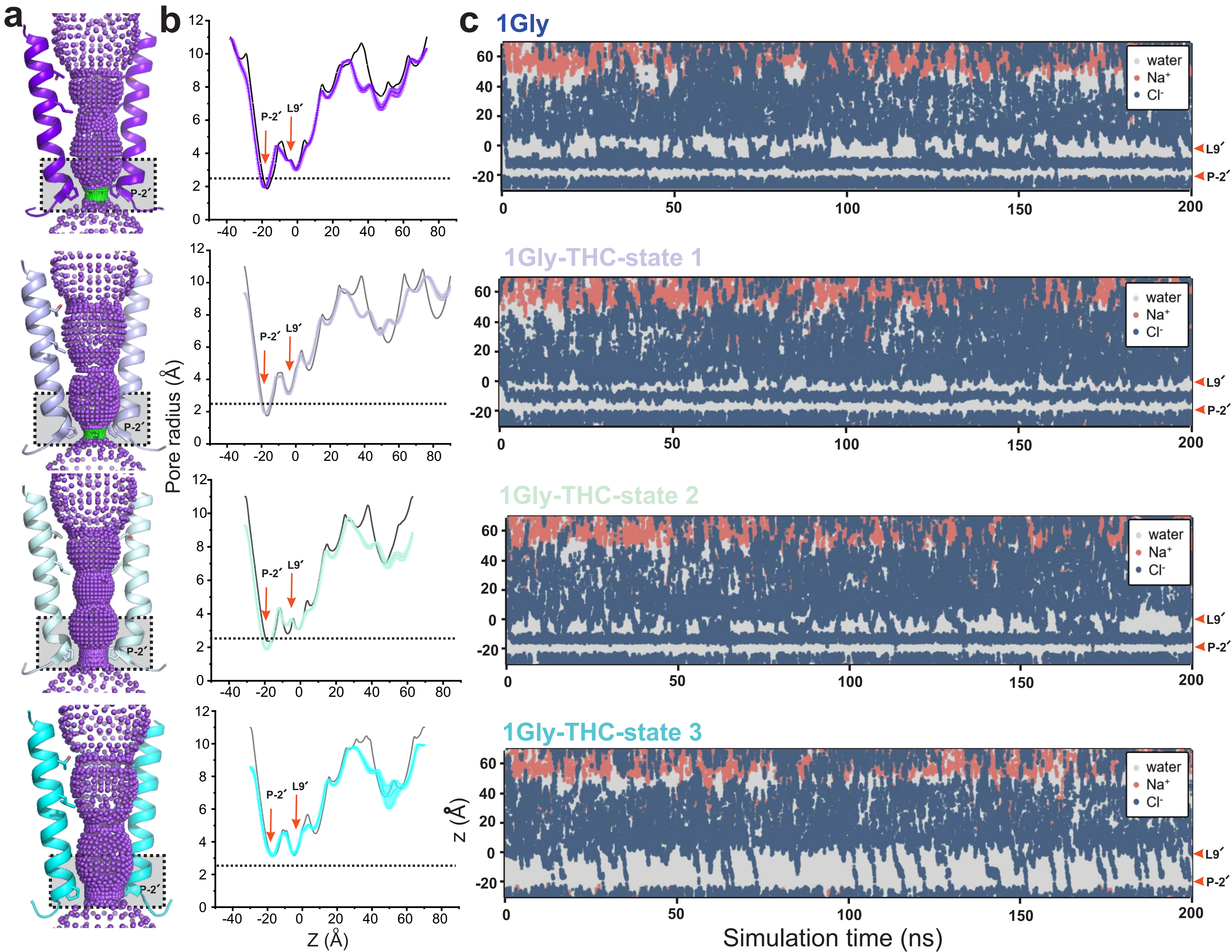
a
b
c
1Gly
1Gly-THC-state 1
1Gly-THC-state 2
1Gly-THC-state 3
P-2´
L9´
Pore radius (Å)
Z (Å)
water
Na+
Cl-
z (Å)
Simulation time (ns)

Supplementary Figure 12. Assessment of conductance state of GlyR-1gly and GlyR-1gly-THC structures (a) Ion permeation pathway along the M2 helices for GlyR-1gly and GlyR-1gly-THC (States 1, 2, and 3). Only two diagonal M2 helices are shown for clarity. Gray box is shown to highlight the constriction at Pro-2′ position. (b) Mean pore radius and one-standard deviations from three independent 30 ns equilibrium simulations for GlyR–Apo structure along the central pore axis. (c) Simulation trajectories along the pore (*z*)-axis of water molecules and chloride ion coordinates in the presence of a +500 mV transmembrane potential difference. The energetic barriers due to the ring of Leu9′ and Pro-2′ are at z ~0 and −20 Å, respectively.

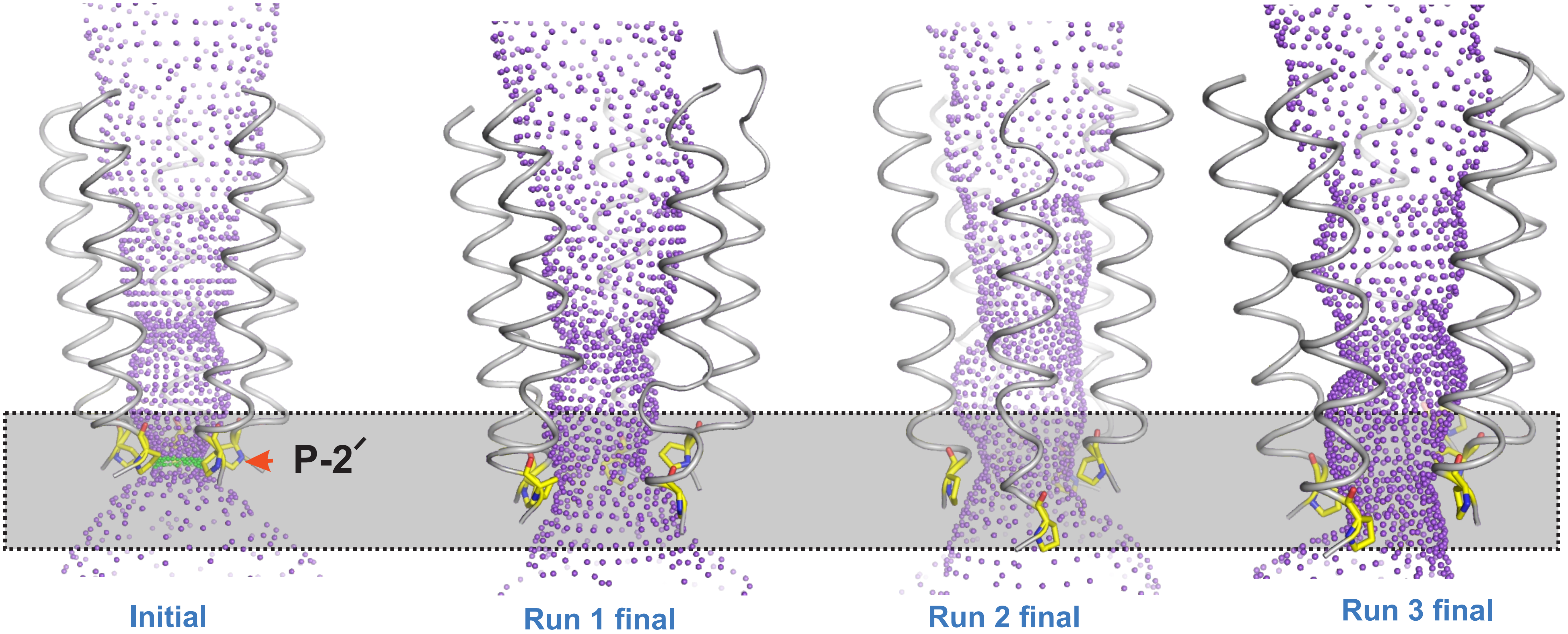

0.1Gly-Docked THC

Supplementary Figure 13. Geometry of the pore during the molecular dynamics simulations. HOLE transmembrane pore profiles for 0.1Gly-Docked THC states from MS simulation runs. The pore profile represents starting conformation (*initial, left*) and final conformations from three in dependent simulation runs. The gray box is shown to highlight the de-pinching of Pro-2′

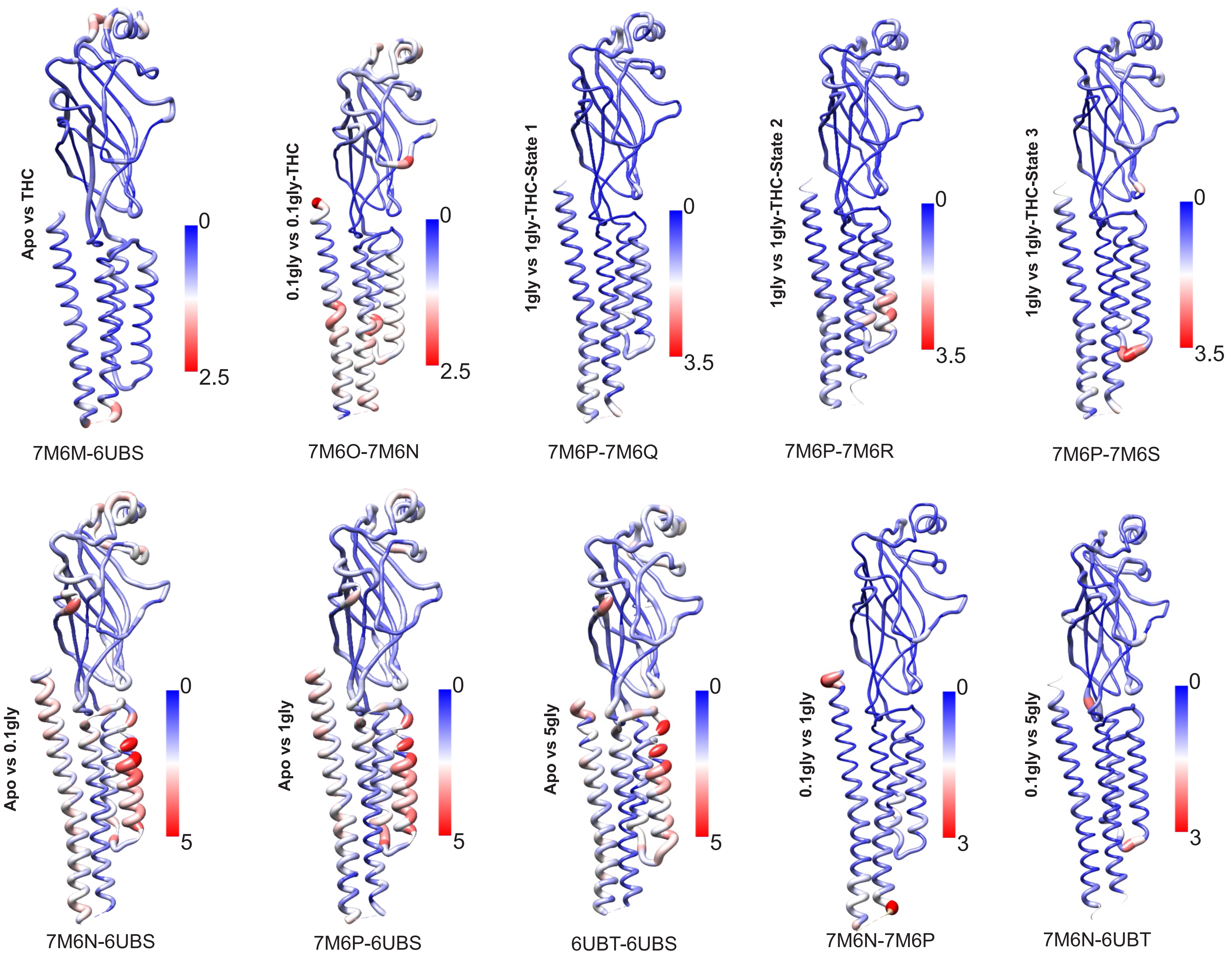
Apo vs THC
0
2.5
7M6M-6UBS
0.1gly vs 0.1gly-THC
0
2.5
7M6O-7M6N
1gly vs 1gly-THC-State 1
0
3.5
7M6P-7M6Q
1gly vs 1gly-THC-State 2
0
3.5
7M6P-7M6R
1gly vs 1gly-THC-State 3
0
3.5
7M6P-7M6S
Apo vs 0.1gly
0
5
7M6N-6UBS
Apo vs 1gly
0
5
7M6P-6UBS
Apo vs 5gly
0
5
6UBT-6UBS
0.1gly vs 1gly
0
3
7M6N-7M6P
0.1gly vs 5gly
0
3
7M6N-6UBT

Supplementary Figure 14. Putty representations of pairwise deviations for the various GlyR receptor conformations. The selection used for superimposition, and the two conformations used, are noted for each image. Single subunit from each pentamer was used for 3D alignment and only backbone C-alpha RMSD was used for calculation. The RMSD color code and tube thickness scale are presented next to each other.

Supplementary Table 1. Sequence of pCS2-a1 plasmid encoding zebrafish GlyRα1 for expression in *Xenopus laevis* and primers used for mutagenesis.

MFALGIYLWETIVFFSLAASQQAAARKAASPMPPSEFLDKLMGKVSGYDARIRPNFKGP
PVNVTCNIFINSFGSIAETTMDYRVNIFLRQQWNDPRLAYSEYPDDSLDLDPSMLDSIWK
PDLFFANEKGANFHEVTTDNKLLRISKNGNVLYSIRITLVLACPMDLKNFPMDVQTCIM
QLESFGYTMNDLIFEWDEKGAVQVADGLTLPQFILKEEKDLRYCTKHYNTGKFTCIEAR
FHLERQMGYYLIQMYIPSLLIVILSWVSFWINMDAAPARVGLGITTVLTMTTQSSGSRAS
LPKVSYVKAIDIWMAVCLLFVFSALLEYAAVNFIARQHKELLRFQRRRRHLKEDEAGDG
RFSFAAYGMGPACLQAKDGMAIKGNNNNAPTSTNPPEKTVEEMRKLFISRAKRIDTVSR
VAFPLVFLIFNIFYWITYKIIRSEDIHKQ

S320A_Fwd, CTTCTCTTCGTCTTCGCTGCCCTGCTGGAGTATG

S320A_Rev, CATACTCCAGCAGGGCAGCGAAGACGAAGAGAA G

W263F_Fwd, ATTGTCATTTTGTCTTTCGTGTCCTTCTGG

W263F_Rev, CCAGAAGGACACGAAAGACAAAATGACAAT

F266A_Rev, GTCCATGTTGATCCAGGCGGACACCCAAGACAA

F266A_Fwd, TTGTCTTGGGTGTCCGCCTGGATCAACATGGAC

W267F_Fwd, TCTTGGGTGTCCTTCTTCATCAACATGGACGC

W267F_Rev, GCGTCCATGTTGATGAAGAAGGACACCCAAGA

P274A_Rev, AACCCCACACGGGCTGCGGCAGCGTCCATGTT

P274A_Fwd, AACATGGACGCTGCCGCAGCCCGTGTGGGGTT

F418A_rev, GAGGAAGACCAGCGGAGCGGCCACACGCGACAC

F418A_fwd, GTGTCGCGTGTGGCCGCTCCGCTGGTCTTCCTC

Supplementary Table 2. Codon optimized zebrafish GlyRα1 sequence used for protein production.

>GlyRalpha1_codon_optimised
ACTAGTATGTTCGCCCTGGGTATCTACCTGTGGGAAACCATCGTGTTCTTCTCCCTGG
CTGCTAGCCAGCAGGCTGCTGCTCGCAAGGCCGCTTCCCCTATGCCTCCCAGCGAAT
TCCTGGACAAGCTGATGGGCAAGGTGTCCGGCTACGACGCTCGCATCCGTCCCAACT
TCAAGGGTCCACCTGTGAACGTCACTTGCAACATCTTCATCAACTCTTTCGGCTCAAT
CGCCGAGACTACCATGGACTACAGGGTGAACATCTTCCTGAGACAGCAGTGGAACG
ACCCACGTCTGGCTTACTCTGAATACCCTGACGACTCACTGGACCTGGACCCCTCTA

TGCTGGACTCAATCTGGAAGCCAGACCTGTTCTTCGCCAACGAGAAGGGCGCTAACT
TCCACGAAGTGACCACTGACAACAAGCTGCTGAGGATCTCCAAGAACGGAAACGTG
CTGTACAGCATCAGAATCACCCTGGTCCTGGCCTGCCCTATGGACCTGAAGAACTTC
CCCATGGACGTCCAGACCTGCATCATGCAGCTGGAGTCCTTCGGTTACACTATGAAC
GACCTGATCTTCGAGTGGGACGAAAAGGGTGCTGTGCAGGTGGCTGACGGACTGAC
CCTGCCTCAGTTCATCCTGAAGGAGGAAAAGGACCTGCGCTACTGCACTAAGCACT
ACAACACCGGAAAGTTCACTTGCATCGAGGCTCGCTTCCACCTGGAACGTCAGATG
GGTTACTACCTGATCCAGATGTACATCCCCAGCCTGCTGATCGTGATCCTGTCCTGG
GTCAGCTTCTGGATCAACATGGACGCTGCTCCAGCTAGGGTGGGTCTGGGCATCACC
ACTGTCCTGACTATGACCACTCAGTCCAGCGGCTCTAGAGCTTCACTGCCCAAGGTG
TCCTACGTCAAGGCCATCGACATCTGGATGGCTGTGTGCCTGCTGTTCGTCTTCAGC
GCCCTGCTGGAGTACGCCGCTGTGAACTTCATCGCTCGCCAGCACAAGGAACTGCTG
CGTTTCCAGCGCCGTAGGAGACACCTGAAGGAGGACGAAGCTGGAGACGGAAGGTT
CTCTTTCGCCGCTTACGGCATGGGACCAGCCTGCCTGCAGGCTAAGGACGGAATGGC
CATCAAGGGTAACAACAACAACGCTCCTACCTCAACTAACCCTCCTGAGAAGACCG
TGGAGGAAATGCGCAAGCTGTTCATCTCTAGGGCCAAGAGAATCGACACTGTGTCA
CGTGTCGCTTTCCCTCTGGTCTTCCTGATCTTCAACATCTTCTACTGGATCACCTACA
AGATCATCCGCTCCGAAGACATCCACAAGCAGCTGGTTCCGCGTGGTAGTCATCACC
ATCACCATCACCATCACTAAGGTACC

Supplementary Table 3 Cryo-EM data collection, refinement and validation statistics.

Sample	GlyR-THC	GlyR-0.1gly-THC	GlyR-0.1gly
PDBid	7M6M	7M6O	7M6N
EMDB id	23700	23702	23701
Data Collection and processing			
Microscope and location	FEI Titan Krios, Frederick National Laboratory for Cancer Research	SLAC	FEI Titan Krios, Case Western Reserve University
Magnification	81000	130000	81000
Voltage	300	300	300
Data collection mode	super-resolution	super-resolution	super-resolution
Camera	K3	K3	K3
Physical pixel size	1.08 Å/pixel	0.68 Å/pixel	1.1 Å/pixel
Defocus range (uM)	−1.0 to −2.0	−1.0 to −2.0	−0.8 to −1.8
Number of movie	5760	5803	7844
Dose per frame	1.45 e-/Å2	1.25	0.85
Number of frames/movie	40	50	70
Initial particle number	124240	190380	147734
Final particle number	22238	29664	89791
Symmetry	C5	C5	C5
Resolution (unmasked, Å)	3.53 Å	3.4 Å	3.11 Å
Resolution (masked, Å)	3.09 Å	2.84 Å	2.61 Å
Map resolution range *	2-8	2-8	2-8
Map sharpening B-factor ($Å^2$)	-30	-30	-30
Refinement			
Initial model used (PDB code)	6UBS	6UBS	6UBS
Composition			
Protein residues	1720	1785	1805
Non Hydrogen atoms	14190	14670	14700
Glycan (NAG) (molecule)	10	5	5
Glycine (molecule)	0	5	5
TCI	5	5	0
Bonds (RMSD)			
Length (Å) (# > 4σ)	0.008(0)	0.009(0)	0.009(0)
Angles (°) (# > 4σ)	1.327(5)	1.374(30)	1.276(0)
Ramachandran plot (%)			
Outliers	0	0	0
Allowed	3.71	3.1	1.68
Favored	96.29	96.9	98.32
Rotamer outliers (%)	0	0	0
Molprobity score	1.41	1.43	1.26
Molprobity clashscore	3.69	5.24	4.89
* Local resolution range	2-8Å	2-8Å	2-8Å

Supplementary Table 4 Cryo-EM data collection, refinement and validation statistics.

Sample	GlyR-1gly-THC			GlyR-1gly
PDBid	7M6Q	7M6R	7M6S	7M6P
EMDB id	23704	23705	23706	23703
Data Collection and processing	State1	State2	State3	
Microscope and location	FEI Titan Krios, Case Western Reserve University			FEI Titan Krios, Case Western Reserve University
Magnification	81000			81000
Voltage	300			300
Data collection mode	super-resolution			super-resolution
Camera	K3			K3
Physical pixel size	1.1 Å/pixel			1.1 Å/pixel
Defocus range (uM)	−1.0 to −2.0			−1.0 to −2.0
Number of movie	4336			5822
Dose per frame	1.2			0.85
Number of frames/movie	40			70
Initial particle number	323908			189243
Final particle number	77813	44653	18156	29483
Symmetry	C5	C5	C5	C5
Resolution (unmasked, Å)	3.5 Å	4.2 A	4.33 Å	3.82 Å
Resolution (masked, Å)	2.91 Å	3.57 Å	3.61 A	3.28 Å
Map resolution range *	2-8	2-8	2-8	2-8
Map sharpening B-factor (Å^2)	-30	-30	-30	-30
Refinement				
Initial model used (PDB code)	6UBS	6UBS	6UBS	6UBS
Composition				
Protein residues	1795	1755	1785	1795
Non Hydrogen atoms	14725	14300	14600	14650
Glycan (NAG) (molecule)	5	0	0	5
Glycine (molecule)	5	0	0	5
TCI	5	0	0	0
Bonds (RMSD)				
Length (Å) (# > 4σ)	0.008(0)	0.008(0)	0.009(0)	0.008(0)
Angles (°) (# > 4σ)	1.152(10)	1.201(0)	1.403(27)	1.266(5)
Ramachandran plot (%)				
Outliers	0	0	0	0
Allowed	3.1	3.47	3.1	3.1
Favored	96.9	96.53	96.9	96.9
Rotamer outliers (%)	0	1	2	0
Molprobity score	1.5	1.45	1.53	1.46
Molprobity clashscore	5.7	4.44	6.25	5.18
* Local resolution range	2-8Å	2-8Å	2-8Å	2-8Å

Supplementary References

1. Swint-Kruse, L. & Brown, C.S. Resmap: automated representation of macromolecular interfaces as two-dimensional networks. *Bioinformatics* **21**, 3327-8 (2005).
